# Destructive Giant Cell Tumor With a Secondary Aneurysmal Bone Cyst of Cervical Spine: A Rare Pediatric Case Report

**DOI:** 10.7759/cureus.23303

**Published:** 2022-03-18

**Authors:** Sidi Mamoun Louraoui, Fadwa Fliyou, Rita Ait Benhamou, Abdessamad El Azhari

**Affiliations:** 1 Neurosurgical Department, Mohammed VI Hospital, Casablanca, MAR

**Keywords:** pediatric, cervical spine, secondary, aneurysmal bone cyst, giant cell tumor

## Abstract

Spinal giant cell tumor (GCT) and aneurysmal bone cyst (ABC) are infrequent neoplasms of bone. Thirty percent of ABCs are secondary to tumors, such as GCT. We report a rare case of a pediatric cervical spine secondary ABC to GCT that had to be multimodally managed through anterior and posterior surgical approach, embolization, and denosumab treatment leading to a stabilization of the remnant. The case shows the importance of therapeutic strategy decision that depends on the patient and the lesion.

## Introduction

Spinal giant cell tumor (GCT) and aneurysmal bone cyst (ABC) are infrequent primary neoplasms of bone [1,2]. GCT is a locally aggressive benign neoplasm. The vertebral localization is rare. They are associated with a large biological spectrum ranging from latent benign to highly recurrent and occasionally metastatic malignant potential [2].

ABC are osteolytic, aggressive, and expansive lesions that account for 2.5% of adult bone tumors. ABCs fall into 30% secondary to other tumors, such as osteoblastoma, giant cell tumor (GCT), hemangioma, osteosarcoma, chondroblastoma, and fibrous dysplasia [3].

Data concerning spinal ABCs secondary to GCT in the pediatric population remains poor as only two cases were found in the literature. Treatment remains poorly understood and recurrences high [4]. We report a rare case of a pediatric cervical secondary ABC.

## Case presentation

**Figure 1:**
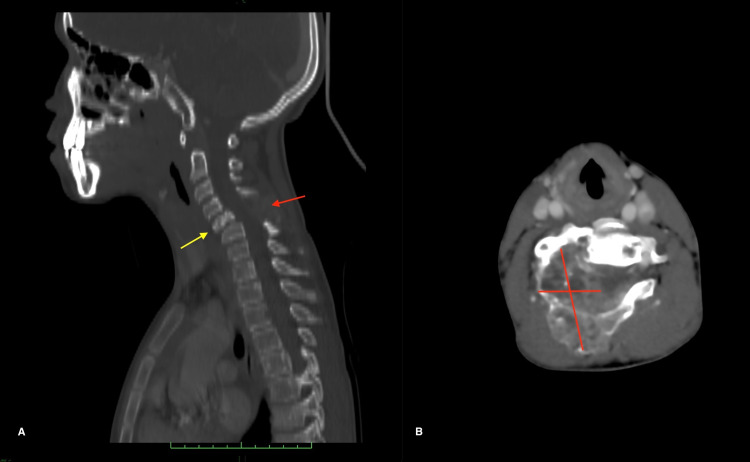
Sagittal (A) and axial injected CT scan (B) showing the heterogeneous lytic mass arising from C3 to C5 lamina (red arrow), compacting C5 body with cervical kyphosis (yellow arrow), encasing the right vertebral artery with preserved flow

An 11-year-old male presented with spontaneous right torticollis evolving for two weeks with radicular pain on the right arm. The neurological examination was in favor of a spinal syndrome and bilateral Babinski without any deficit. Computed tomography scan showed a heterogeneous lytic mass arising from C3 to C5 lamina (2.5x5.2x3.5 cm), compacting C5 body with cervical kyphosis, encasing the right vertebral artery with the preserved flow (Figure 1).

**Figure 2:**
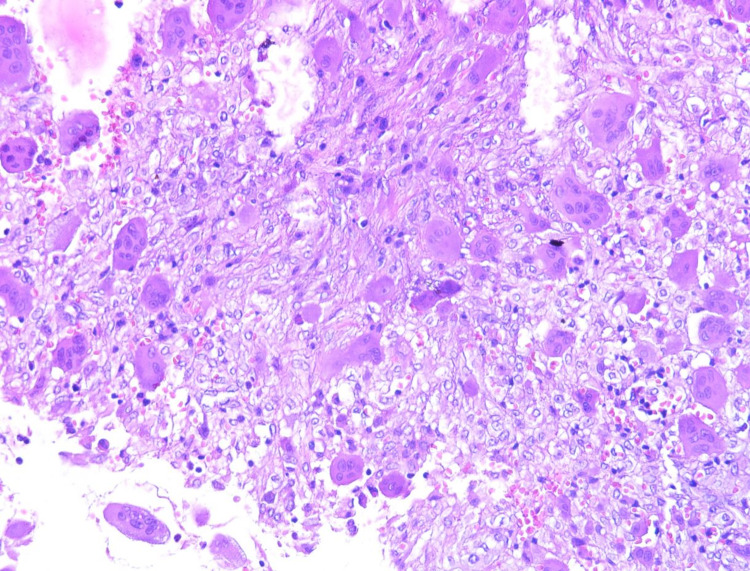
Histological aspect showing a giant cell with multiple nuclei, on mononuclear stroma and telangiectasis vascular aspect

The patient was planned for a staged surgery with adjuvant chemotherapy: an anterior approach for stabilization, and then posterior excision of the lesion, medullar decompression, and stabilization. An anterior approach was performed first. Unfortunately, due to tumor bleeding, excision of the anterior portion and C5 resection had to be aborted. C4-C6 fixation was performed with moderate kyphosis reduction with a stabilization goal. The immediate postoperative course was simple. Pathology was in favor of a giant cell tumor with a secondary aneurysmal bone cyst component (Figure 2).

**Figure 3:**
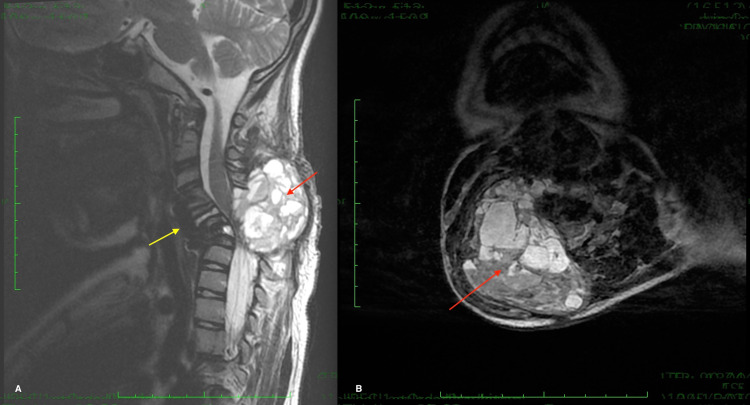
Sagittal (A) and axial (B) MRI showing posterior tumor growth with spinal cord compression, right paravertebral soft tissue extension (red arrow), and the anterior fixation by C4-C6 plate and cervical kyphosis (yellow arrow)

Adjuvant chemotherapy with denosumab was indicated prior to posterior excision of the lesion and anterior approach to restore a proper cervical lordosis. The patient presented just before chemotherapy with diffuse swelling of the neck with right paravertebral mass and tetraparesis. Imaging was in favor of posterior tumor growth (4.5x6.3x5.6 cm) with a worsening of the posterior spinal cord compression and right paravertebral soft tissue extension. No worsening of the cervical kyphosis was noted on the imaging (Figure 3).

A primary embolization of the lesion with posterior decompression and fixation of the spine was performed. The lesion was soft, with moderate bleeding, which allowed a 90% resection of the lesion, decomposing the spinal cord with a remnant around the right vertebral artery. The patient totally recovered from his neurological deficit and benefited after recovery from denosumab therapy.

**Figure 4:**
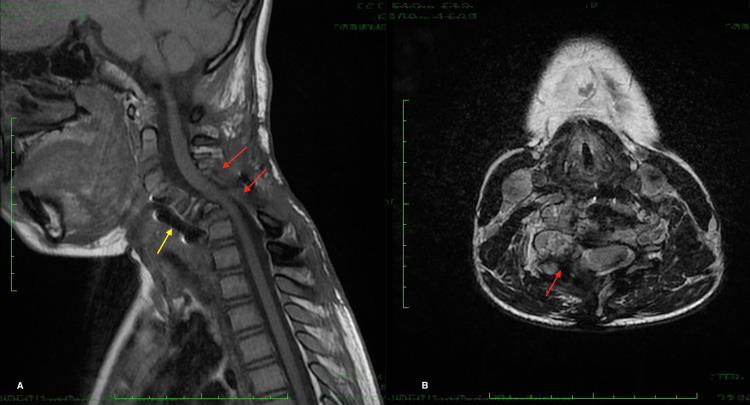
Sagittal (A) and axial (B) postoperative MRI showing posterior decompression (red arrow) with cervical kyphosis (yellow arrow)

**Figure 5:**
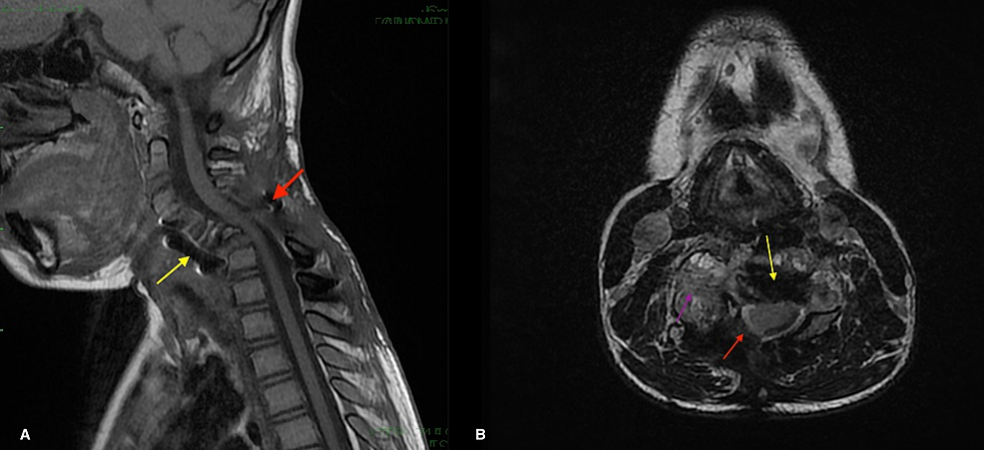
T1 sagittal (A) and T2 axial MRI at two years showing a stabilized lesion, spinal cord decompression (red arrow), a remnant around the vertebral artery (purple arrow), and anterior cervical plate (yellow arrow)

An anterior approach to restoring cervical lordosis was indicated, but the patient refused the surgery. Follow-up at two years showed a stabilization of the patient's neurological status (no deficit), and imaging at two years showed a stabilized lesion with a remnant around the right vertebral artery (Figures 4-5).

## Discussion

GCT are rare benign but locally aggressive tumors that account for 4-10% of bone lesions. The vertebral location is atypical and represents 2.7-7%. They are named after their histological aspect: the shape of nodules of osteoclast-like giant cells that express the receptor activator of nuclear factor kappa B ligand (RANKL), which is an essential mediator for osteoclast survival [2,5].

ABC is also benign and aggressive bony lesions [3,4]. Histologically they have a soft fibrovascular core that contains cystic-like cavities filled with blood surrounded by a bone shell [1,4]. Many theories have been reported in the literature concerning the genesis of ABCs, such as a local circulatory disturbance, improper repair of traumatic injuries leading to a subperiosteal hemorrhage, or a hemorrhage on a preexisting pathology [3,4].

Wang et al. published the largest series of adult spinal secondary ABC. Out of the 33 patients included in the study, 20 were secondary to GCT, making this association the most frequent one (60,6%) [3]. The cervical spine is the least common location for spinal ABCs, accounting for only 2% of cases. Pediatric patients are at a greater risk of developing ABCs, and nearly 80% appear in the first two decades of life [3,4].

GCT, as well as ABC, affect the posterior elements of the vertebrae in 75% of the cases, with an anterior potential extension. It has been reported that the laminae, pedicles, and the spinous process of the spine are the first affected structures [1,4].

Although ABC is higher in the pediatric population, secondary ABCs remain rare. In 2017, Protas et al. published a review and reported only one patient with ABC secondary to GCT of the cervical spine [4]. Like GCT, secondary ABC arising from the spine requires an aggressive surgical resection to achieve local tumor control [1,3,4]. Otherwise, it exposes the patient to higher risks of recurrence. The therapeutic options on the cervical spine, especially in the pediatric population, depend on the location of the lesion, the symptoms, the patient's age, and the degree of encasement of the vertebral artery. Complete surgical resection with subsequent stabilization if needed is the guarantee of recovery. Nineteen patients over 71 in the Protas study recurred; 11 of them benefited from subtotal resection of the lesion [4]. Series have shown that resection with wide margins has been associated with fewer recurrence rates (0-16%) [2]. 

Pharmaceutical treatments have been introduced to treat non-surgical patients or the impossibility of gross total removal. The current understanding of GCT's molecular biology and the role of RANK/RANKL pathway explains the interest in using denosumab [2]. It is a human monoclonal antibody that bonds specifically to the cytokine RANKL preventing it from activating the RANK receptor, thus inhibiting osteoclast function [2,6]. It also has been proven that ABC shows some similarities explaining the high frequency of the association and giving hope concerning the effects of denosumab despite the actual lack of protocol or recommendations for this use [2,6,7]. 

Kurucu et al. described nine patients treated by denosumab for ABC. Two patients benefited from surgical resection for large tumors after the treatment. Four patients recurred at 10 and 24 months [6,8]. Dürr et al. on nine patients showed local control on all patients with two classified tumor-free [6,9]. On the other hand, they draw attention to the patient's age and the complications (disturbed growth and dental development) in young children [6].

On the other hand, arterial embolization of the lesion is another therapeutic option for ABC treatment. Considering that cervical ABC's vascularization is high, embolization is often used as an adjuvant treatment to reduce vascular supply to the lesion, justifying our use during the recurrence. Protas et al. considered four patients for exclusive embolization of the lesion that recurred and required either re-embolization or secondary surgical treatment [4].

## Conclusions

Cervical secondary ABC to GCT is rare and very challenging in the pediatric population. Therapeutic management might need a staged and multimodal strategy that needs to be adapted to the lesion and the patient.
